# Temperature control board design and validation for skipper-CCD sensors using a buck converter

**DOI:** 10.1016/j.ohx.2025.e00703

**Published:** 2025-09-19

**Authors:** Marcelo Barrientos, Martin Barrientos, Jorge Rodas, Alfredo Renault, Carlos Romero, Fabian Palacios, Claudio Chavez, Ana Martina Botti

**Affiliations:** a CITHED, Department of Electronic and Mechatronic Engineering, Facultad de Ingenieria, Universidad Nacional de Asuncion, Luque 110948, Paraguay; b Laboratory of Distributed Systems, Facultad de Ingenieria, Universidad Nacional de Asuncion, Luque 110948, Paraguay; c Laboratory of Mechanics and Energy, Facultad de Ingenieria, Universidad Nacional de Asuncion, San Lorenzo 111421, Paraguay; d Fermi National Accelerator Laboratory, Batavia, IL 60510, USA

**Keywords:** Temperature control board, Skipper-CCD, Buck converter, Raspberry Pi, OSCURA experiment, Printed circuit board design

## Abstract

This article describes the creation and validation of a custom temperature control board explicitly designed for Skipper-CCD sensors. The board is versatile and can be used in various experimental setups. It consists of two galvanically isolated sections: a control section equipped with a Raspberry Pi and essential instrumentation for measurement and protection, and a power section with a buck converter and additional instrumentation for enhanced protection and monitoring. The seamless integration of these sections provides robust temperature control and comprehensive safeguards against potential issues. Through careful design and extensive experimental validation, the developed board ensures precise thermal management tailored to the unique needs of Skipper-CCD sensors. Its effectiveness has been demonstrated in the OSCURA experiment and can serve as a model for potential applications in other projects.

## Graphical abstract

**Figure:**
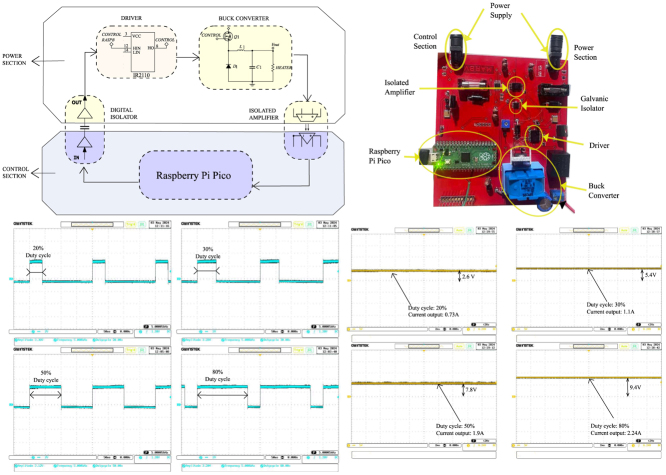

## Specifications table

**Table:** 

Hardware name	*Temperature control board for Skipper-CCD sensors*
Subject area	•*Electronic engineering* •*Power electronics* •*Power converters*

Hardware type	•*Signal measurements and sensors* •*Power electronics* •*Electrical engineering*

Closest commercial analogue	*The custom temperature control board for Skipper-CCD sensors can be closely compared to the Lake Shore temperature controller. Lake Shore controllers are commonly used in scientific and research applications for accurate temperature measurement and control. They employ PID control algorithms and autotuning features to ensure stable temperatures.*

Open source license	GNU General Public License (GNU GPL v3)

Cost of hardware	US$ 250

Source file repository	*Source files repository* (OSF) *write the DOI URL here*. http://dx.doi.org/10.17605/OSF.IO/VRN2E

## 1 Hardware in context

The temperature control system for the Skipper CCD sensor represents a highly specialised design that integrates a buck converter mounted on a custom-engineered circuit board. This system is systematically divided into two stages, separated by galvanic isolation, to ensure the precision and stability required for ultra-low-noise applications. Unlike previous implementations, this work provides a detailed quantitative characterisation of the performance, reproducibility, and system alignment with state-of-the-art solutions, addressing a critical gap in the literature.

The first control stage is centred on a Raspberry Pi, the core processing unit that executes the control algorithms. This stage incorporates temperature, current, and voltage sensors to monitor the system’s operating conditions. This level of precision is comparable to or exceeds that of commercially available temperature controllers, such as those described in [1]. The design of the control stage ensures that the system can meet the delicate requirements of high-sensitivity experiments, as highlighted in [2] on the sensitivity of Skipper CCD.

A key feature of the system is the implementation of galvanic isolation between the control and power stages [3]. This isolation ensures that low-power control signals generated by the Raspberry Pi remain unaffected by electrical noise or interference from the power electronics. By preserving the integrity of the control signals, the system enables accurate temperature regulation, which is essential for maintaining the performance of the Skipper CCD sensor during long-duration experiments. Even minor temperature deviations can significantly impact the accuracy of the data, as discussed in [4] in the context of developing low-noise CCDs.

The second power stage comprises a buck converter that regulates power transmission to the heating element. The converter achieves efficiency under typical operating conditions, with advanced protection mechanisms that ensure stable performance across a wide range of input voltages (5–24 V). Precision measurement capabilities enable the system to maintain power delivery accuracy, which is crucial for achieving the thermal stability necessary for high-precision measurements. These performance metrics are thoroughly documented, enabling reproducibility and facilitating further optimisation by the scientific community. The design principles align with those outlined in [5], which is foundational work on power electronics.

Previous generations of Skipper CCD temperature controllers [6], such as those deployed at Fermilab, have demonstrated successful operation in research environments. However, these implementations lack detailed documentation of their design and operational characteristics. The existing literature provides valuable information on the performance of these controllers; however, it does not offer a comprehensive breakdown of their architecture and components. This work addresses this gap by presenting a thorough description of the Skipper CCD temperature controller, including quantitative performance data and a detailed comparison to state-of-the-art solutions, as emphasised in [7] in the context of open-source hardware development.

The system’s design is particularly relevant for experiments such as OSCURA, where temperature fluctuations can significantly impact data accuracy [8]. By providing an open-source, reproducible, and thoroughly characterised solution, this work not only advances the field of temperature control for high-sensitivity scientific instrumentation but also sets a new standard for transparency and accessibility in hardware development. Including performance metrics, reproducibility guidelines, and comparative analysis ensures that this paper holds significant scientific merit and provides a valuable resource for future research.

## 2 Hardware description

### 2.1 Design and components

The temperature control board is carefully designed to ensure precise thermal management for Skipper-CCD sensors. This is essential for low-noise, high-sensitivity experiments, such as OSCURA. The design priorities integrate high-efficiency components while maintaining a compact and robust form factor to meet the strict requirements of these advanced sensors.

The board’s power management circuitry is designed to distribute power effectively to different system sections, ensuring each component receives a stable and appropriate voltage. This circuitry includes protection features, such as safeguards against overvoltage and overcurrent conditions, which are essential for the board’s reliable operation under various experimental conditions. Key components include:

**Fig. 1:**
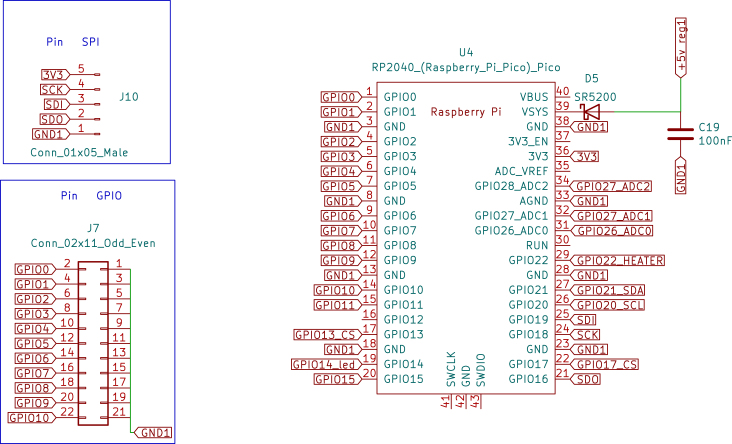
Schematic diagram of the Raspberry Pi Pico.

**Fig. 2:**
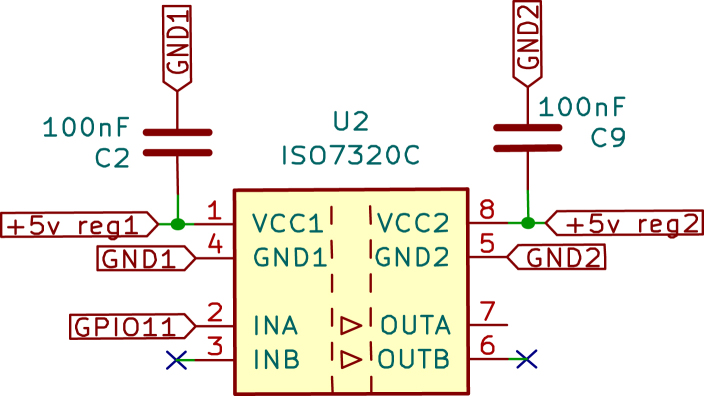
Digital isolator.

**Fig. 3:**
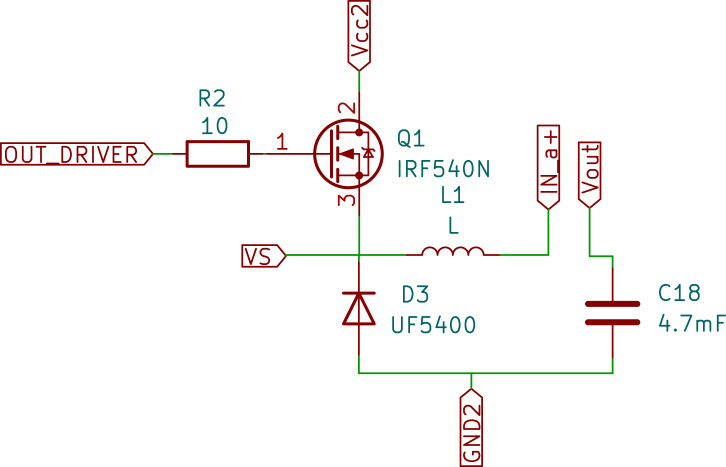
The MOSFET in the buck converter.

**Fig. 4:**
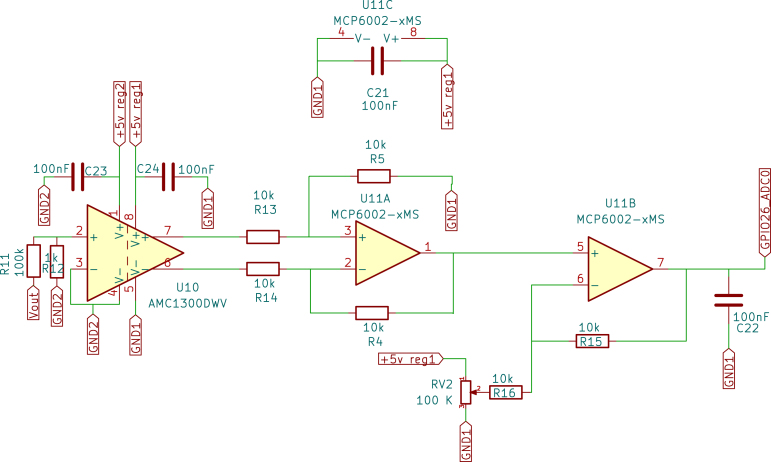
Isolated operational amplifier circuit.

• Raspberry Pi Pico: The Raspberry Pi Pico is a versatile and powerful microcontroller developed by the Raspberry Pi Foundation. It is built around the RP2040 chip, designed specifically for the Pico, making it a robust and adaptable solution for many embedded applications. Below are the key technical specifications, capabilities, and specific applications in the temperature control board for the Skipper-CCD sensors.
  – Technical Specifications:
    * Processor: The Raspberry Pi Pico is powered by the RP2040 microcontroller chip, which features a dual-core Arm Cortex-M0+ processor running at a clock speed of up to 133 MHz. This provides ample processing power for handling complex control algorithms and real-time processing tasks.
    * Memory: The microcontroller has 264 kB of SRAM and 2 MB of onboard flash memory. This memory configuration is sufficient for most embedded applications, including those requiring real-time data processing and storage of control algorithms.
    * Operating Voltage: The Pico operates at a nominal voltage of 3.3 V, with acceptable input voltages from 1.8 V to 5.5 V. This flexibility enables easy integration into various circuits and compatibility with multiple sensors and peripherals.
    * I/O Capabilities: The Raspberry Pi Pico provides 26 multi-function GPIO pins, which can be configured for various functions, including digital input/output, Pulse-Width Modulation (PWM), I2C, SPI, UART, and ADC. This extensive I/O capability enables the Pico to interface with a wide range of components, making it adaptable to various project requirements.
    * Power Consumption: The Pico is designed with low power consumption in mind. It features several power-saving modes that can be leveraged in energy-sensitive applications. Its ability to operate at low power levels makes it ideal for battery-powered or energy-efficient designs.
    * Programming and Development: The Raspberry Pi Pico is programmable via MicroPython, C/C++, and other languages, with an extensive ecosystem of libraries and tools available. This ease of programming, combined with the Pico’s powerful hardware, makes it a popular choice among hobbyists and professionals.
  – Types of Applications: Due to its flexibility, the Raspberry Pi Pico is widely used in various applications. It is commonly employed in embedded systems where real-time processing and control are required. Its low power consumption and wide range of I/O options make it suitable for Internet of Things (IoT) applications. The Pico is also ideal for controlling motors, relays, and other devices in automated systems. Furthermore, due to its ease of use and affordability, it is frequently utilised in educational environments to teach programming and electronics.
  – Application in Temperature Control Board: In the context of the temperature control board for Skipper-CCD sensors, the Raspberry Pi Pico plays a central role in managing the system’s control logic, Fig. 1. The specific application of the Pico in this project includes:
    * Temperature Monitoring: The Pico is responsible for continuously monitoring the temperature readings from high-precision sensors integrated into the system. These readings provide crucial information to ensure the Skipper-CCD sensors operate within their optimal temperature range.
    * Power Adjustment: Based on the temperature data, the microcontroller adjusts the power delivered to the Skipper-CCD through the buck converter. However, this adjustment follows a predefined control strategy rather than a closed-loop feedback system. The power levels are modulated using GPIO pins configured for PWM output to set the buck converter’s switching frequency and duty cycle.
    * Interfacing with Sensor Network: The Pico interfaces with the network of sensors and other components on the board, coordinating their operation for effective thermal management. Several GPIO pins are used for this purpose:
      · GPIO26 (ADC0): Connected to the temperature sensor for reading analogue temperature data.
      · GPIO27 (ADC1): Additional analogue input for temperature data or other sensor readings.
      · GPIO15 and GPIO16: Configured as SPI interface pins to communicate with other components, such as the digital isolator or additional peripheral devices that require high-speed data transmission.
      · GPIO17 and GPIO18: Used for UART communication to send data to external devices or receive commands for operational adjustments.
    * Power Management: The Pico helps regulate power delivery to the Skipper-CCD sensors to optimise system efficiency. This is particularly important in maintaining the precision required in experiments like OSCURA, where stable power conditions are necessary to avoid temperature fluctuations that could impact experimental outcomes.
• Digital Isolator: The Digital Isolator is essential to the temperature control board, providing robust galvanic isolation between the control and power sections. This separation is crucial for ensuring the safety and integrity of the low-voltage control electronics, such as the Raspberry Pi Pico, from potential high-voltage surges and electrical noise generated by the power section.
  – Component Details:
    * Type and Model: The digital isolator used in this design is the ISO7320. This high-performance, dual-channel digital isolator is well-suited for applications requiring reliable signal isolation and protection [9].
    * Encapsulation Type: The ISO7320 comes in a Small Outline Integrated Circuit (SOIC)-8 package (with eight pins), which is compact and suitable for designs where space is critical. The SOIC package provides good thermal performance and easy integration into Printed Circuit Board (PCB) layouts.
    * Channels: The ISO7320 offers two independent isolation channels, simultaneously isolating two signals. This dual-channel capability is ideal for separating critical control signals in systems where multiple signal paths require isolation.
    * Isolation Method: The ISO7320 utilises capacitive isolation technology. This method utilises capacitive coupling to transmit signals across an isolation barrier, thereby maintaining electrical separation between the input and output. Capacitive isolation is known for its reliability, high-speed performance, and ability to withstand high voltage differences across the barrier.
    * Bandwidth: The isolator features a high data transmission rate with a bandwidth of up to 25 Mbps, ensuring that control signals are transmitted with minimal delay and high fidelity. This high-speed performance is crucial in applications where timing accuracy is critical, such as in PWM control for power converters.
    * Isolation Rating: The ISO7320 provides isolation up to 3000 Vrms, offering excellent protection against high-voltage surges that could otherwise damage sensitive control electronics. This high level of isolation ensures the safety and longevity of the components on both the control and power sides of the circuit.
  – Application in This Project: In the temperature control board designed for Skipper-CCD sensors, the ISO7320 digital isolator plays a crucial role in maintaining the integrity of the control signals, Fig. 2. The ISO7320 isolates the low-voltage control signals generated by the Raspberry Pi Pico from the high-voltage power signals that control the buck converter. This ensures that any high-voltage transients or noise generated by the power section do not affect the microcontroller or other sensitive electronics.
• Gate Driver: The IR2110 is a high-voltage, high-speed MOSFET and IGBT driver that can independently drive both high- and low-side power switches. This driver is crucial in applications that require fast and efficient switching, such as the buck converter used in this project.
  – Key Features:
    * Floating high-side driver with bootstrap operation, allowing control of high-side MOSFETs in a half-bridge configuration.
    * Undervoltage lockout (UVLO) protection to prevent malfunctioning in low-voltage conditions.
    * Propagation delay matching to ensure synchronised switching between high-side and low-side MOSFETs.
  – Application in this Project: In this system, the IR2110 drives the IRF540N MOSFET in the buck converter, enabling efficient power switching while ensuring isolation from the control signals. The IR2110 receives the PWM signals from the Raspberry Pi Pico, which are first isolated by the ISO7320, providing robust and noise-free operation in a high-voltage environment.
• MOSFET Power: The IRF540N is an N-channel power MOSFET widely used in power electronics applications due to its high current-carrying capacity and low on-resistance. It is packaged in a TO-220 case, which provides robust heat dissipation and durability. Key Specifications:
  – Voltage Ratings:
    * Drain-to-source voltage (Vds): 100 V
    * Drain-to-gate voltage (Vdg): 100 V
    * Gate-to-source voltage (Vgs): ±20 V
  – Current Ratings:
    * Maximum continuous drain current (Id): 33 A at 25 °C
    * Maximum continuous drain current (Id): 23 A at 100 °C
  – Power Dissipation: 150 W (max)
  – Thermal Resistance: 62.5 °C/W junction-to-ambient
  – Switching Characteristics:
    * Turn-on delay time (td(on)): 9.5 ns
    * Rise time (tr): 57 ns
    * Turn-off delay time (td(off)): 40 ns
    * Fall time (tf): 55 ns
  – Application in This Project: The IRF540N drives the buck converter, Fig. 3, essential for managing the power delivered to the Skipper-CCD sensors. The MOSFET operates as a high-speed switch controlled by the IR2110 driver, ensuring efficient switching and stable power regulation. The fast switching times and low on-resistance of the IRF540N contribute to efficient power conversion, minimising losses and maintaining precise sensor temperature control. The MOSFET’s ability to handle high currents ensures the system can deliver the necessary power without overheating or experiencing performance degradation. In conjunction with the digital isolator and driver circuitry, the IRF540N provides reliable and efficient control over the power delivered to the Skipper-CCD sensors, ensuring that they operate within their specified temperature range for optimal performance.
• Power Inductors: The TDK common mode choke was used as the inductor due to its market availability, providing 15 mH of inductance and a maximum current rating of 3 A. Tests showed a saturation current of 4.2 A, sufficient for the maximum design current (3 A). The inductance remained stable (±10%) up to 15 kHz, well within the converter’s operating range(5 kHz).
• Operational amplifiers (Op-Amps) The MCP6001 operational amplifier is a key component in various general-purpose applications that require low power consumption and precision. The following is a detailed description of the MCP6001 Op-Amp, its technical characteristics, and its application in the Skipper-CCD temperature control system.
  – Technical Characteristics:
    * Gain Bandwidth Product: 1 MHz, which provides a moderate speed suitable for general-purpose applications.
    * Rail-to-Rail Input/Output: The op-amp supports full-range input and output swings, making it ideal for low-voltage operation.
    * Supply Voltage: Operates from a single supply voltage as low as 1.8 V up to 6.0 V, making it compatible with various power systems.
    * Supply Current: 100 μA (typical), ensuring low power consumption, particularly useful in battery-powered systems.
    * Operating temperature range: −40 °C to ＋125 °C, which makes it suitable for industrial environments.
  – Applications:
    * Photodiode amplifiers: The MCP6001 is commonly used to amplify low-current signals from sensors such as photodiodes.
    * Analogue Filters: It is an excellent choice for designing low-pass or band-pass filters.
  – Configuration in the Temperature Control Board: In this temperature control system, the MCP6001 op-amp is used in a cascaded configuration to achieve precise signal amplification and noise reduction [10].
    * Differential amplifier stage: The first stage is configured as a differential amplifier to measure the difference between two input signals. This configuration is ideal for rejecting common-mode noise, which is crucial when working with small signals in noisy environments, such as those found in power electronics.
    * Inverter amplifier stage: The second stage is configured as an inverter amplifier. This stage corrects the signal’s phase as the differential amplifier inverts it. The signal is re-inverted by cascading the inverting amplifier after the differential amplifier Fig. 4, thus recovering the original signal phase. This two-stage configuration ensures that the signal is processed without any phase inversion, which is critical for maintaining the integrity of the control loop.
• Buck converter: The buck converter is a fundamental component of the temperature control board, responsible for efficiently regulating the power supplied to the Skipper-CCD sensors. It steps down the input voltage to a controlled output voltage, ensuring stable system operation while minimising power dissipation. The buck converter used in this design consists of the following key components previously detailed:
  – Switching MOSFET: (IRF540N) Serves as the main power switch.
  – Gate Driver: (IR2110) Provides fast and efficient switching control.
  – Diode: (Schottky SR5200) Provides a freewheeling path for inductor current during switching.
  – Capacitors: Electrolytic reduce voltage ripple and stabilise output.
  The buck converter operates using PWM, where the Raspberry Pi Pico generates a control signal that modulates the duty cycle of the MOSFET. The stored energy in the inductor continues to power the load. The Schottky diode provides a current path, preventing interruptions. This switching process repeats at a frequency of 5 kHz, maintaining a regulated voltage at the output while achieving high efficiency (>90%).
  – Applications of Buck Converters: Buck converters are widely used in various applications, including:
    * Consumer Electronics: Used IoT devices to step down battery voltage efficiently.
    * Automotive Systems: Power regulation in electric vehicles (EVs), infotainment systems, and adaptive LED headlights.
    * Renewable Energy: Solar charge controllers use buck converters to step down voltage from solar panels to battery-compatible levels.
    * Industrial Automation: Applied in Programmable Logic Controllers (PLCs) and embedded systems for a stable DC voltage supply.
    * Telecommunications: Used in base stations and network routers to power RF circuits with precise voltage control.
  – Application in This Project: In the temperature control board, the buck converter is specifically designed to:
    * Regulate power delivery to the Skipper-CCD sensors, maintaining a stable supply voltage.
    * Minimise heat dissipation, increasing system efficiency compared to linear regulators.
    * Enable precise control using PWM signals from the Raspberry Pi Pico, allowing dynamic adjustment of power levels.

**Fig. 5:**
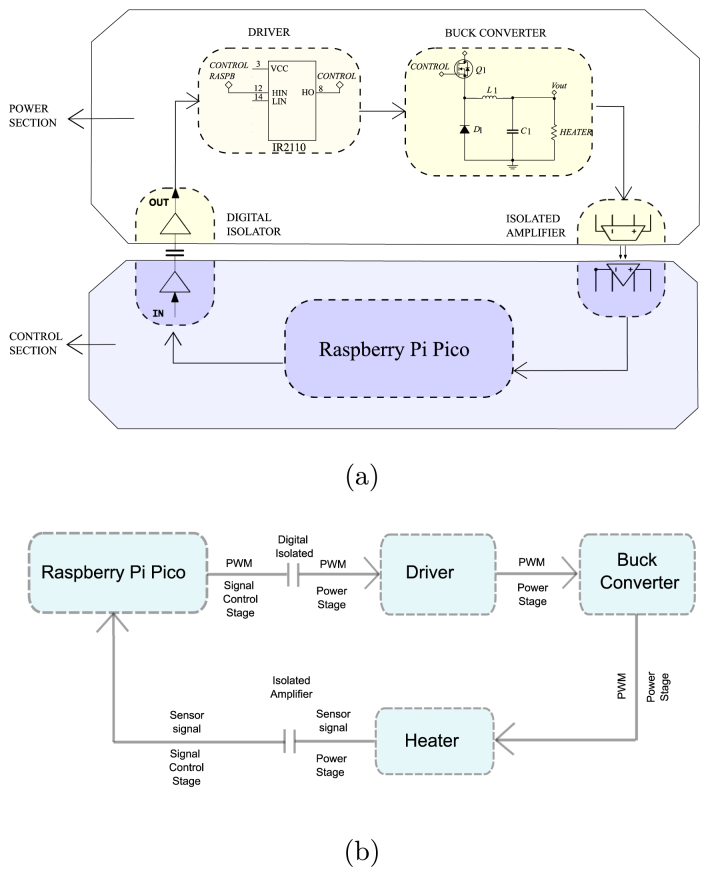
Overview of the Skipper-CCD sensor system. (a) Schematic diagram showing the main components and signal paths involved in the operation of the Skipper-CCD sensors. (b) Control-oriented block diagram illustrating the system structure in terms of inputs, control actions, feedback, and outputs.

### 2.2 System integration

The integration of these components is carefully engineered to strike a balance between performance, efficiency, and reliability. The Raspberry Pi Pico microcontroller serves as the central hub, orchestrating the interaction between the temperature sensors and the buck converter (Fig. 5).

• Temperature control loop: The control loop is managed by the Raspberry Pi Pico, which reads the temperature data from the sensors and adjusts the output of the buck converter accordingly. This feedback loop is designed to respond quickly to temperature fluctuations, ensuring that the Skipper-CCD sensors remain within their optimal operating temperature range.
• Signal Conditioning and Feedback: Operational amplifiers are integrated into the signal path to condition the sensor output before sending it to the microcontroller. This setup reduces noise and ensures the control system receives accurate temperature data, which is critical for precise control.
• Power Distribution: Power management circuitry is designed to distribute power efficiently across the board, with the buck converter providing the primary voltage to the sensors and the microcontroller. The system minimises power losses and ensures all components operate within the specified parameters.

**Fig. 6:**
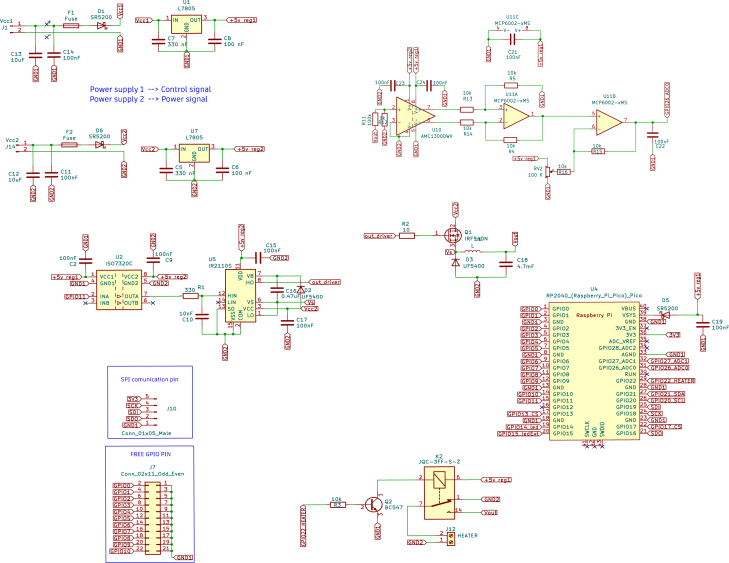
Complete schematic of the temperature controller designed in KiCad.

**Fig. 7:**
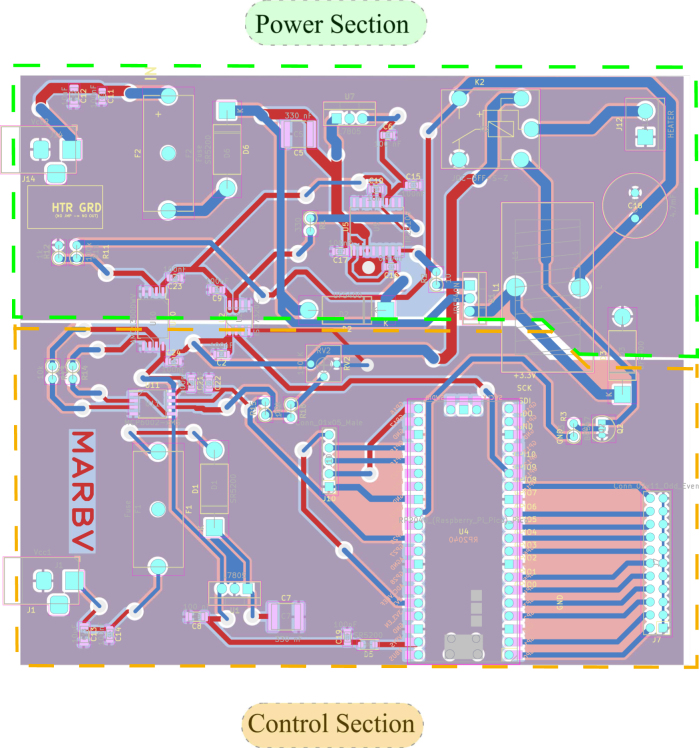
Final assembly of the PCBs of the designed circuits.

Fig. 7 shows the main designed motherboard. This motherboard has six slots for voltage and current conditioning circuits, which can be inserted interchangeably, as the slots on the main board and the conditioning circuits have identical power and signal connections. The main motherboard combines the other modular plates designed and mentioned above [11] (see Fig. 6).

### 2.3 Galvanic isolation

Galvanic isolation is a crucial design consideration for the temperature control board, particularly given the sensitivity of the Skipper-CCD sensors and the potential for interference from the power supply.

• Isolation Techniques: The board uses optocouplers and isolated DC-DC converters to electrically separate the control and power sections. This isolation prevents noise from the power section from affecting the control logic and sensor readings, which is critical for maintaining the integrity of the temperature control loop.
• Benefits of Isolation: By implementing galvanic isolation, the board protects the sensitive Skipper-CCD sensors from potential electrical interference and enhances the system’s overall reliability. This design approach ensures that the control signals remain clean and that the sensors can operate in their low-noise, high-sensitivity mode without disruption.

The galvanic isolator, a key component in the temperature control system for the Skipper-CCD sensor, electrically separates the power stage from the control stage, thereby creating two distinct ground planes: GND1 for the control stage and GND2 for the power stage. This separation is critical to prevent electrical noise and interference from the high-power components from affecting the sensitive control electronics.

The specific component used to achieve this galvanic isolation is ISO7320. The ISO7320 is a high-performance, dual-channel digital isolator that utilises silicon dioxide (SiO2) insulation to provide electrical isolation. It is designed to transfer digital signals across an isolation barrier, ensuring no direct electrical connection between the input and output sides.

In this temperature control system, the PWM signals generated by the Raspberry Pi Pico in the control stage are fed into the ISO7320 isolator. The ISO7320 transmits these signals across the isolation barrier while maintaining electrical isolation between the two stages. This ensures the control signals remain unaffected by any noise or voltage spikes from the power stage.

By creating separate ground planes (GND1 and GND2), the ISO7320 effectively isolates the low-voltage control circuitry from the high-voltage power components. This isolation is essential for protecting the control stage, including the Raspberry Pi Pico and various sensors, from potential damage and interference, thereby ensuring the overall reliability and precision of the temperature control system. The isolated signals are then adapted in voltage by the IR2110 driver, which controls the MOSFET of the buck converter in the power stage, enabling precise thermal management for the Skipper-CCD sensor.

### 2.4 Pulse width modulation trigger signal adaptation stage

The proposed design meets the system’s needs in terms of bandwidth and protection. The PWM pre-actuation stage is a critical subsystem of the coupling circuit. Its function is to convert the voltage levels of the digital output ports of the Raspberry Pi Pico control module, whose logic levels vary between 0 and 3.3 V, to the appropriate activation levels required by the transistor in the buck converter configuration. The transistor handles a floating voltage in a buck converter, necessitating proper voltage level adaptation.

This voltage level adaptation is achieved using the IR2110 driver. The IR2110 can convert the low-voltage PWM signals from the Raspberry Pi Pico to the higher voltage levels required by the transistor. This driver ensures the transistor is actuated correctly, enabling it to handle the necessary power levels for efficient buck converter operation [12].

The design employs the IR2110 driver to ensure that the control signals from the Raspberry Pi Pico are accurately translated to the required activation voltages for the transistor. This adaptation meets the system’s bandwidth requirements. It provides essential protection to the control stage, ensuring reliable and robust operation of the entire temperature control system for the Skipper-CCD sensor. This capability is crucial for maintaining stable and precise thermal management, particularly in high-precision experiments like those conducted in the OSCURA project.

### 2.5 Potential research applications of the proposed hardware

• The control board can be adapted to stabilise the temperature of a wide range of sensitive detectors (e.g., CCDs, photodiodes, infrared sensors) where thermal noise reduction is critical. The design offers a documented and cost-effective solution that enables other groups to replicate precise temperature control without relying on closed or proprietary systems.
• The platform serves as a testbed for evaluating advanced control techniques in real hardware, which may benefit research groups exploring modern control strategies.
• Its modular structure (buck-boost converter, microcontroller, galvanic isolation) makes it suitable for training in power electronics and for rapid prototyping of laboratory thermal stabilisation systems.

## 3 Design files summary

**Table:** 

Design filename	File type	Open source license	Location of the file
*Control board*	*PCB file and Gerber, KiCad*	*GNU GPL v3*	https://osf.io/apuze
*Control schematic*	*Schematic file, pdf*	*GNU GPL v3*	https://osf.io/w6uve
*List of components*	*List of Components and prices file*	*GNU GPL v3*	https://osf.io/kw8xp
*Control Project*	*Project file, KiCad*	*GNU GPL v3*	https://osf.io/b83kt
*Control schematic*	*Schematic file, KiCad*	*GNU GPL v3*	https://osf.io/a34fm
*Firmware*	*Source code*	GNU GPL v3	https://osf.io/vrn2e/files/osfstorage

• *Temperature control board: Contains the KiCad project files for the temperature control PCB and the Gerber file to produce the PCB.*
• *Temperature control schematic.pdf: Schematic or electronic diagram of temperature control components.*
• *List of Components and prices.csv: List of components of the temperature control components.*
• *Temperature control project: Contains the KiCad project files for the main motherboard PCB and the main motherboard Gerber file to produce the PCB.*
• *Temperature control schematic: Contains the KiCad schematic or electronic diagram of the main motherboard components.*

## 4 Bill of materials summary

**Table:** 

Designator	Component	Value	Voltage/Spec	Number	Per unit (US$)	Total (US$)	Source
C1, C4, C6–C8, C11, C13–C15, C19, C20	Ceramic X7R 0805	100 nF	50 V	11	0.155	1.705	Mouser
C2, C9	Ceramic X7R 2220	330 nF	100 V	2	0.470	0.940	Mouser
C3, C10	Ceramic X7R 0805	100 nF	50 V	2	0.155	0.310	Mouser
C5, C12	Tantalum C	10 µF	16 V	2	1.600	3.200	Mouser
C16	Electrolytic	4.7 mF	25 V	1	2.620	2.620	Mouser
C17	Ceramic NP0 0603	0.47 µF	50 V	1	0.920	0.920	Mouser
C18	Ceramic X7R 0402	10 nF	50 V	1	0.370	0.370	Mouser
D1, D2, D5	Schottky	SR5200	200 V, 5 A	3	0.580	1.740	Mouser
D3, D4	Ultrafast	UF5400	400 V, 3 A	2	0.450	0.900	Mouser
F1, F2	PTC fuse	1.6 A	30 V	2	1.190	2.380	Mouser
J1, J2	Power connector	Vcc1, Vcc2	–	2	2.450	4.900	Mouser
J3	Male header	1 × 5	–	1	0.930	0.930	Mouser
J4	Pin header	2 × 11	–	1	1.190	1.190	Mouser
J5	HEATER connector	–	–	1	4.850	4.850	Mouser
K1	Relay	JQC-3FF-S-Z	10 A, 250VAC/30VDC	1	4.850	4.850	Mouser
L1	Inductor	100 µH	2 × 15 mH 3 A	1	3.670	3.670	Mouser
Q1	MOSFET	IRF540N	100 V, 33 A	1	4.300	4.300	Mouser
Q2	BJT	BC547	45V, 100 mA	1	0.350	0.350	Mouser
R1, R4–R6, R9	Resistor 0805	10 kΩ	1%, 0.125 W	5	0.710	3.550	Mouser
R2	Resistor 0805	100 kΩ	1%, 0.125 W	1	0.710	0.710	Mouser
R3	Resistor 0805	1 kΩ	1%, 0.125 W	1	0.350	0.350	Mouser
R7	Resistor 1206	10 Ω	1%, 0.25 W	1	0.900	0.900	Mouser
R8	Resistor 0805	330 Ω	1%, 0.125 W	1	0.710	0.710	Mouser
RV1	Trimmer	100 kΩ	0.5 W	1	1.200	1.200	Mouser
U1, U4	Regulator	L7805CV	35 V max, 5 V output	2	0.580	1.160	Mouser
U2	OpAmp	MCP6002	5.5 V max, 1 MHz	1	0.290	0.290	Mouser
U3	Isolator	AMC1300	5.5 V max, ±250 mV input	1	4.260	4.260	Mouser
U5	Digital isolator	ISO7320C	3–5.5 V	1	2.790	2.790	Mouser
U6	Driver	IR2110S	600 V, 2 A	1	4.140	4.140	Mouser
U7	Microcontroller	RP2040 Pico	1.8V–5.5 V	1	19.500	19.500	Mouser

## 5 Build instructions

The Temperature Control Board for Skipper CCD Sensors follows a dual-stage architecture with galvanic isolation, using the ISO7320C isolator to separate power and control domains. This approach minimises ground loop interference and preserves signal integrity in low-noise analogue sections.

The board employs a hybrid assembly methodology:

• Surface-Mount Technology (SMD) for signal conditioning and control circuitry.
• Through-Hole Technology (THT) for high-power components (>3 A continuous current).

This combination ensures optimal power density, electrical performance, and mechanical robustness.

### 5.1 Fabrication process

The PCB was manufactured by PCBWay, using Gerber/X2 files exported from KiCad 7.0 [13].

Fabrication Steps:

1. Obtain the manufacturing files from the OSF repository (Gerber_TemperatureControl.zip).
2. Configure the PCB manufacturing request at PCBWay with the following parameters:
  • Dielectric: FR4 Tg130 ($\epsilon_{r}=4.5$ @1 MHz)
  • Copper: 1oz (35 μm) electrodeposited
  • Finish: Lead-Free HASL (3–5μSn coating)
  • Tolerance: ±0.1mm mechanical outline
3. Submit the design for fabrication and wait for PCBWay to complete the process.
4. Upon receiving the PCBs, perform a visual inspection and a continuity test using a multimeter to check for:
  • Short circuits or broken traces
  • Correct pad alignment based on the schematic in KiCad

### 5.2 Assembly process

Once the PCB is verified, the assembly process follows a sequential soldering approach to minimise errors and improve efficiency.

**Fig. 8:**
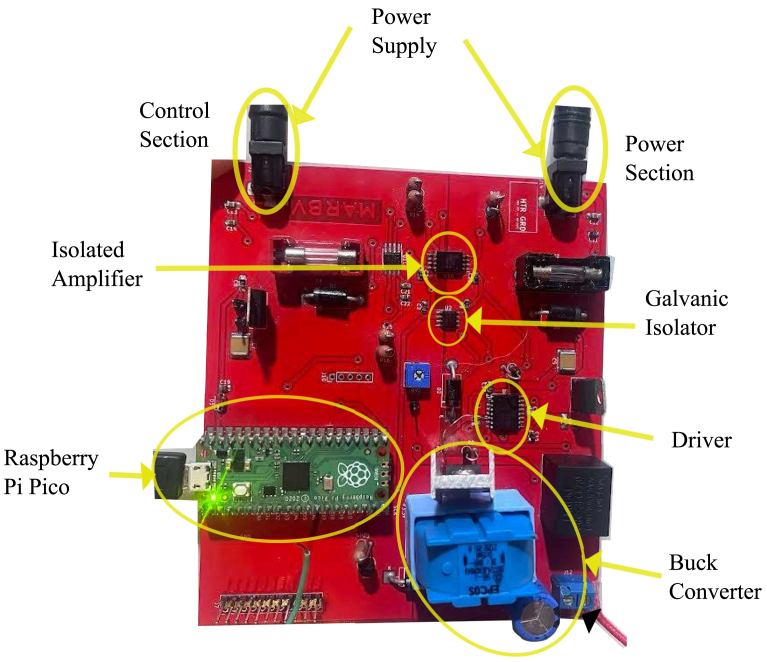
Fully assembled thermal control.

Step 1: Gather Components & Tools Bill of Materials (BOM): Ensure all components listed in the repository are available. Tools Required:
  • Soldering station with closed-loop thermal control (320 °C ± 5 °C)
  • Hot air rework station (for SMD components)
  • ESD Tweezers (for precise handling of 0402 SMD components)
  • Multimeter (for continuity checks)
  • Oscilloscope (for PWM and voltage signal validation)
Step 2: Soldering Process (Ordered by Component Size)
  • Phase 1: SMD Components (Smallest to Largest)
    – Start with capacitors and resistors (0402, 0603 sizes).
    – Solder ICs and active components (e.g., ISO7320C, MCP6001 Op-Amps).
    – Reflow soldering process for fine-pitch components using a hot air station.
    – Verify each connection before proceeding to the next step.
  • Phase 2: Through-Hole Components (THT) and High-Power Devices
    – Place and solder larger components, such as MOSFETs (IRF540N), connectors, and inductors.
    – Ensure proper heatsinking (apply thermal paste and attach heatsinks to MOSFETs).
    – Secure power connectors and relays using mechanical fasteners where necessary.
  • Phase 3: Final Inspection & Electrical Tests
    – Perform a final continuity check before powering the board.
    – Check isolation resistance between control and power stages.
    – Power up the board and test the output voltage, ensuring the buck converter operates correctly (see Fig. 8).

### 5.3 Verification and debugging

Functional Tests Before Deployment:

• Power-on test: Ensure all voltage rails provide correct output.
• Temperature control validation: Use an oscilloscope to verify PWM control.
• Final system test: Simulate real operating conditions and verify stability.

This structured approach ensures a reproducible and error-free fabrication process, addressing manufacturing, assembly, and verification.

## 6 Operation instructions

The following section presents step-by-step operational instructions for using the hardware.

1. Check on the dashboard that the safety switch is in the ON position and the emergency stop button is in the correct position. This safety measure ensures better equipment operation and reduces the chances of human error or accidents.
2. Activate the contactor, check that the power supply is on and verify that the pre-charge activation timer deactivates after 4 s.
3. Connect the computer with a USB cable.
4. Start the programming interface of the Raspberry Pi Pico with Tony.
5. Compile pseudo-code of the control algorithm.
6. Perform experiments.
7. Flip the safety switch back to the OFF position once you are done, and close the interface. **Safety Notice**: High voltage is handled at the input of the voltage transformation board corresponding to the left side of the board. Therefore, precautions should be taken to prevent users from accidental shocks. Do not touch any of the components connected to the high-voltage terminals.

## 7 Validation and characterisation

To validate and characterise the performance of the temperature control system for the Skipper CCD sensor, a series of detailed measurements was conducted using an oscilloscope. These measurements ensured that all system components, from the microcontroller to the final heater temperature regulation, were functioning correctly.

### 7.1 Digital signal verification:

**Fig. 9:**
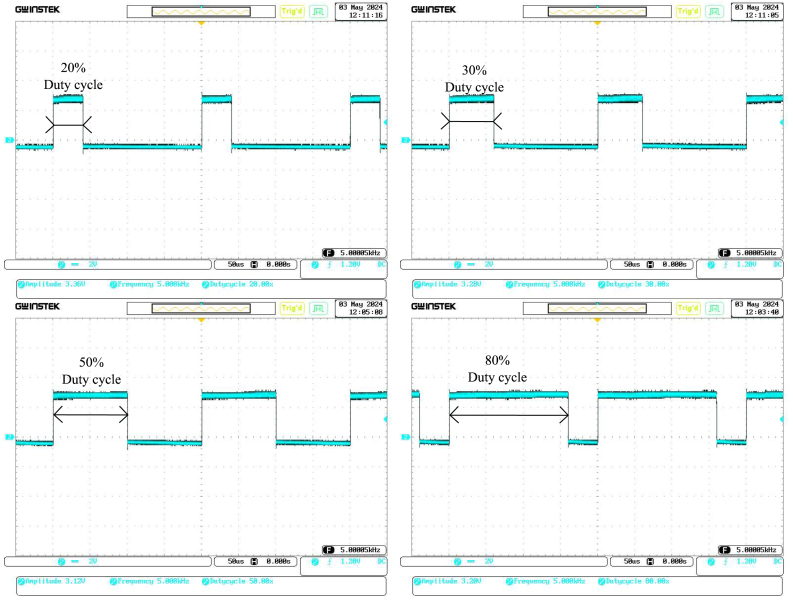
The PWM observed with the oscilloscope.

During the first test, we checked the digital output signals from the Raspberry Pi, with a specific focus on the PWM signal. We programmed the Raspberry Pi to generate a PWM signal to control the power delivered to the heater. The PWM signal was measured using an oscilloscope to verify that it had the correct frequency, duty cycle, and voltage levels. This step was critical to confirm that the microcontroller generated a stable and accurate control signal to drive the following circuit stages (see Fig. 9 and Table 1).

**Table 1:** Characterisation of digital signal.

Duty cycle	Input voltage	Output voltage	Output/Input ratio
20%	12 V	2.6 V	0.12
30%	12 V	5.4 V	0.45
50%	12 V	7.8 V	0.62
80%	12 V	11.6 V	0.96

### 7.2 Driver output measurement

**Fig. 10:**
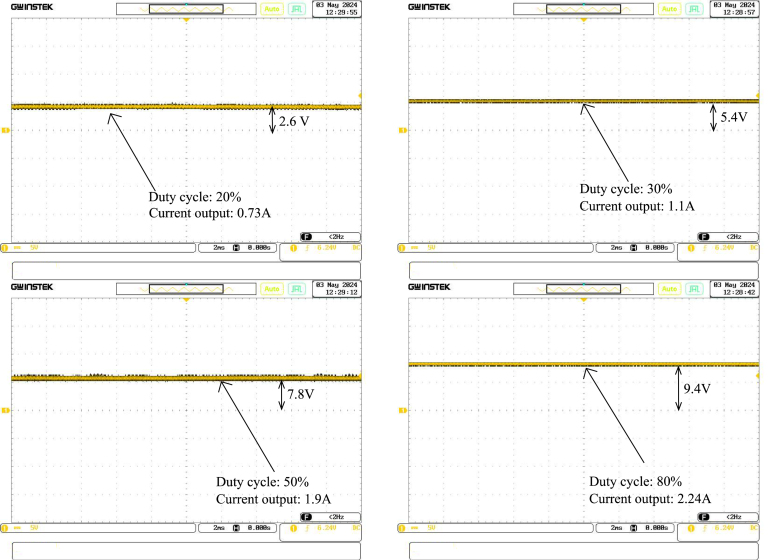
The output observed with oscilloscope.

After confirming the PWM signal from the Raspberry Pi, the next step involved transferring this signal to the driver circuit, which is responsible for conditioning the control signal to manipulate the buck converter appropriately. The oscilloscope was once again utilised to measure the driver’s output voltages, ensuring that it accurately converted the PWM input into a voltage suitable for driving the buck converter. This process ensured the driver delivered the correct voltage range required to control the buck converter’s transistor switching. Fig. 10 shows the output voltage of the buck converter.

### 7.3 Buck converter output verification

The third measurement focused on the output of the buck converter, which is responsible for adjusting the power delivered to the heater. This test aimed to verify that the buck converter was performing as expected by regulating the output voltage in response to the input from the driver. The test involved varying the duty cycles of the PWM signal and measuring the corresponding output voltages with an oscilloscope to confirm that the converter was providing the correct voltage levels required to control the heater. This validation ensured that the buck converter could adjust the power appropriately, delivering a stable voltage in response to the PWM input.

### 7.4 Conclusions

The temperature control system for the Skipper CCD sensor is a highly sophisticated and well-engineered solution designed to meet the stringent requirements of sensitive applications such as those in the OSCURA project. A buck converter, mounted on a custom PCB, is central to the system’s design, effectively managing the power delivered to the heater that regulates the sensor’s temperature. The board’s two-stage architecture, with galvanic isolation separating the control and power stages, is critical in ensuring precision and stability.

The control stage, driven by a Raspberry Pi controller, integrates critical temperature, current, and voltage sensors, enabling real-time feedback and precise adjustments to maintain the required operating conditions. The integration of these sensors, along with the advanced control algorithms executed by the Raspberry Pi, ensures a highly responsive system that can meet the delicate requirements of the Skipper CCD sensor, where even slight deviations in temperature could have significant impacts.

The galvanic isolation between the control and power stages is a crucial feature, preventing interference from the high-power components of the buck converter from affecting the sensitive control electronics. This design enhances the system’s precision, increasing its robustness and reliability, making it well-suited for demanding scientific applications.

In summary, the temperature control system effectively balances power regulation and precision control, providing a stable and controlled environment for the Skipper CCD sensor. This design ensures that the sensor operates under optimal conditions, essential for the success of high-precision experiments in dark matter research, where accurate data collection is paramount. Future work may include a more detailed evaluation of the system behaviour under input voltage variations, as well as an assessment of the ripple level in different operating conditions. This would enable a more comprehensive validation of the filtering stage and its effectiveness in preventing undesired harmonic content that could impact downstream processes.

## CRediT authorship contribution statement

**Marcelo Barrientos:** Writing – original draft, Validation, Software, Methodology, Investigation, Formal analysis. **Martin Barrientos:** Writing – original draft, Validation, Software, Methodology, Investigation, Conceptualization. **Jorge Rodas:** Writing – review & editing, Writing – original draft, Investigation, Funding acquisition, Data curation. **Alfredo Renault:** Writing – original draft, Supervision. **Carlos Romero:** Writing – review & editing, Supervision. **Fabian Palacios:** Validation, Software, Data curation. **Claudio Chavez:** Writing – review & editing, Supervision. **Ana Martina Botti:** Writing – review & editing, Supervision, Conceptualization.

## Declaration of competing interest

The authors declare that they have no known competing financial interests or personal relationships that could have appeared to influence the work reported in this paper.

The author is an Editorial Board Member/Editor-in-Chief/Associate Editor/Guest Editor for this journal and was not involved in the editorial review or the decision to publish this article.
